# Digital Research Environment(DRE)-enabled Artificial Intelligence (AI) to facilitate early stage drug development

**DOI:** 10.3389/fphar.2023.1115356

**Published:** 2023-03-24

**Authors:** Jeffrey S. Barrett, Solmaz Eradat Oskoui, Scott Russell, Amanda Borens

**Affiliations:** Aridhia Digital Research Environment, Glasgow, United Kingdom

**Keywords:** artificial intelligence, drug developement, drug discovery, data sharing, digital research environment

## Abstract

Early-stage drug discovery is highly dependent upon drug target evaluation, understanding of disease progression and identification of patient characteristics linked to disease progression overlaid upon chemical libraries of potential drug candidates. Artificial intelligence (AI) has become a credible approach towards dealing with the diversity and volume of data in the modern drug development phase. There are a growing number of services and solutions available to pharmaceutical sponsors though most prefer to constrain their own data to closed solutions given the intellectual property considerations. Newer platforms offer an alternative, outsourced solution leveraging sponsors data with other, external open-source data to anchor predictions (often proprietary algorithms) which are refined given data indexed upon the sponsor’s own chemical libraries. Digital research environments (DREs) provide a mechanism to ingest, curate, integrate and otherwise manage the diverse data types relevant for drug discovery activities and also provide workspace services from which target sharing and collaboration can occur providing yet another alternative with sponsors being in control of the platform, data and predictive algorithms. Regulatory engagement will be essential in the operationalizing of the various solutions and alternatives; current treatment of drug discovery data may not be adequate with respect to both quality and useability in the future. More sophisticated AI/ML algorithms are likely based on current performance metrics and diverse data types (e.g., imaging and genomic data) will certainly be a more consistent part of the myriad of data types that fuel future AI-based algorithms. This favors a dynamic DRE-enabled environment to support drug discovery.

## Graphical Abstract

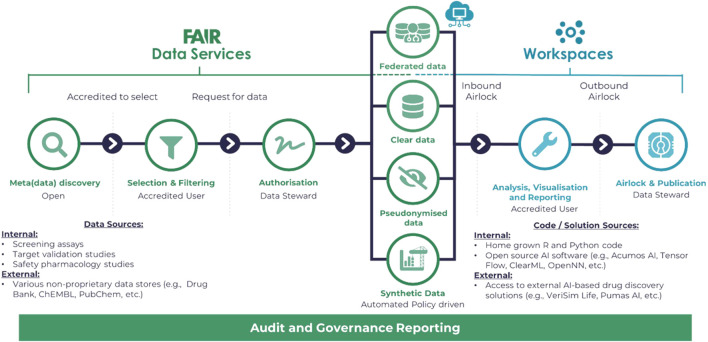

## Introduction

### The drug discovery process and milestones

The discovery phase includes early aspects of drug research designed to confirm pharmacologic targets, identify investigational drug candidates, and perform initial experiments that allow scientists to rank and select candidates for preclinical evaluation. This first stage of the process takes approximately three to 6 years. Researchers hope to identify one or more promising drug candidates for further investigation, ultimately in humans. Investigators conduct studies in cells, tissues, and animal models to determine whether the target can be influenced by a drug candidate. Target validation is crucial to help scientists identify the most promising approaches before going into the laboratory to develop potential drug candidates, increasing the efficiency and effectiveness of the R&D process.

**FIGURE 1:**
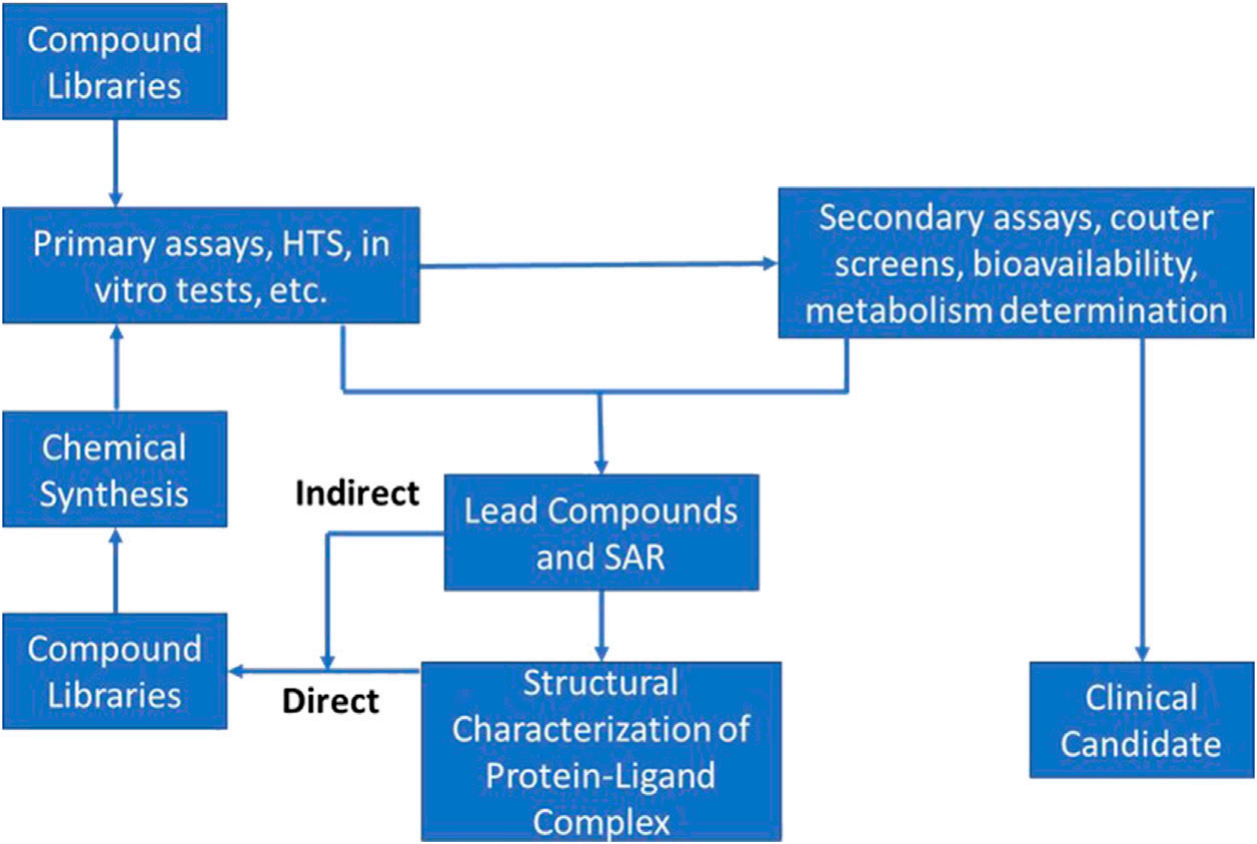
Schematic of typical Discovery phase activities used in the process of target identification and early candidate selection; often referred to and described as the “DMTA cycle” (Design-Make-Test-Analyze).

In actuality this phase consists of two distinct segments: an initial discovery phase, followed by a development phase. These two phases differ significantly from each other with respect to scope, challenges, and approaches. Differences notwithstanding, discovery and development must be integrated into a coherent whole for the process to be successful. Accordingly, much thought has been devoted to insuring scientific, logistical, and organizational aspects of such integration are taken into consideration and optimized. Figure 1 provides a schematic representation of the modern drug discovery process focusing only on the elements critical to target validation and candidate selection. As the current discovery and preclinical phases of development are very much defined by the design and conduct of discrete experiments (e.g., the initial pre-clinical pharmacological screening especially while *in vivo* studies are ongoing), AI/ML approaches can help by both refining these experiments as well as test assumptions related to their outcome. As confidence in this approach improves, this could obviate the need for some of these experiments entirely and limit the amount of *in vivo* animal testing.

The entire process of drug discovery and preclinical development is summarized for the eventual purpose of filling a new drug application, in standardized form of Common Technical Document (CTD), containing five modules (Jordan, 2014). A high-level summary of the process would include diverse data, critical decisions and documentation but this would be true of all drug development phases on the surface. Artificial intelligence (AI) approaches to inform drug development are most focused at early and late stages of development mostly based on the nature and type of data generated in these stages. At early stages of development, the chemical space is generally viewed as comprising > 1,060 molecules (Paul et al., 2021). The virtual chemical space is vast and suggests a geographical map of molecules by illustrating the distributions of molecules and their properties. The idea behind the illustration of chemical space is to collect positional information about molecules within the space to search for bioactive compounds and, thus, virtual screening helps to select appropriate molecules for further testing. Several chemical spaces are open access, including PubChem (PubChem, 2023), ChemBank, DrugBank (Drug Bank, 2023), ChEMBL (ChEMBL, 2023), and ChemDB.

AI is well-suited for these tasks because it can handle large volumes of data with enhanced automation (Bohr and Memarzadeh, 2020). AI involves several method domains, such as reasoning, knowledge representation, solution search, and, among them, a fundamental paradigm of machine learning (ML). ML uses algorithms that can recognize patterns within a set of data that has been further classified. A subfield of the ML is deep learning (DL), which engages artificial neural networks (ANNs). While discovery groups are eager to leverage AL/ML to acquire meaningful insights from the enormous data they hold or acquire, the accuracy of the AI/ML models depends on the volume and quality of the data used as an input for training them. Inaccurate input data can result in misleading outcomes delivered by the AI/ML models but generally this approach is more forgiving with respect to data quality and uncertainty relative to traditional modeling approaches. While automation systems can cleanse data based on explicit programming rules (e.g., imputation algorithms), it’s almost impossible for them to fill in all missing data gaps without some manual intervention or plugging in additional data source feeds. However, machine learning can make calculated assessments on missing data based on its reading of the situation.

The cost of bringing new drugs from bench to bedside has become excessively steep. In identifying these trends, AI/ML-driven *in silico* platforms are alluring to the pharmaceutical and healthcare industry due to their multidimensional, predictive capabilities, and the associated increased efficiency. Traditional model-informed drug development (MIDD) approaches have been used in drug discovery and development over the last 2decades with the recent increase in complexity from the usage of AI/ML-driven *in silico* platforms. Table 1 highlights some of the more common model types and impact on early-phase drug development. Application opportunities for AI/ML can be associated with all stages of drug discovery and development. For example, drug-target validation and engagement, identification of prognostic biomarkers and evaluation of digitized clinical pathology data in clinical trials, and finally high-accuracy predictions of the pharmacokinetic, pharmacodynamic, and efficacy parameters from a limited pool of physiological and pharmacological preclinical and clinical datasets. As each of these decision points also represents a milestone for early phase drug development, one can also appreciate that there are no obvious gaps in an AI-driven approach which seems to coincide with the recent investment in these approaches (Maharao et al., 2020; Paul et al., 2021).

**TABLE 1:** Data, model and stage-gate decision connectivity in a traditional MIDD drug discovery paradigm.

Stage-gate decision	Typical data to inform decision	Complimentary model(s)	Stakeholders
Target validation	Drug discovery data: *in vitro* screening data; chemical libraries; *in silico* modeling results, etc.	QSAR, QSP models	Pharmacology, Medicinal Chemistry
Target indication(s)	Commercial data on medical need, prevalence, etc. (e.g., claims data; epidemiologic data)	HECON; pharmacoeconomic models	R&D Therapeutic areas, Franchise commercial groups
Candidate selection	Preclinical (animal *in vivo*) PK/PD experiments, Toxicology (TK/outcomes) trials, *in vitro* biomarker data, IVIVC data, Human PK/PD, patient RWD	PK, PK/PD, Pop-PK/PD	Pharmacology, Formulations, Toxicology/Safety Assessment, Epidemiology, Clinical Pharmacology,
FIH dose		PBPK, Biopharmaceutic models, QSP models	R&D Therapeutic Areas
Biomarker strategy			
POC trial design			
Endpoint selection			
Patient enrollment criteria			

AI expertise is highly desirable by a multitude of sectors now and AI technologies are being recruited by universities and scientific institutions. As these technologies advance and become more sophisticated, experts are certain that they will revolutionize academia. It has been maintained by some that areas in which AI-enabled technologies will change the game in science and academia are varied and driven by enhanced access to data in an unprecedented speed and volume, peer review procedures that enable data-driven scientific hypotheses and.

AI-enabled technologies that are paving the way for open-source science (Lexis Nexis Report, 2020). As drug development is inherently multidisciplinary, it will be incumbent upon the leadership in the core disciplines of biology, chemistry, engineering, pharmacy and other life and quantitative sciences to offer AI/ML curriculum to its student population in order to better prepare the future workforce and ensure that the supply of core disciplines has the requisite training to appreciate and apply these methodologies.

### Collaborative research environments: Platforms, TREs and DREs

#### Data generation and FAIR

The hurdle to speeding the pace of both patient diagnosis and drug development is that patient health data is often held and accessed by a single group or organization (“silos,” in other words), and patient confidentiality and uncertainty around governance and provenance makes data-sharing problematic. Adding to that aspect is the fact the what constitutes patient level health data is not all maintained in the same operational data store. It is quite common for patient records, pharmacy data and genomic data to reside in different data structures (more silos). To overcome this hurdle, researchers and organizations are leaning into a relatively new method of health data management, by establishing trusted research environments (TREs) (Bacchelli, 2022). TRE is becoming a commonly used acronym among the science and research community. In general, a TRE is a secure collaborative research environment for digital analysis of data. TREs can only be accessed by approved researchers. Governance processes and TRE features ensure only approved researchers can access data, and no data enters or leaves the environment without the express permission of an approved proxy for the data owner. Because data stays put, the risk of patient confidentiality is reduced. However successful the TRE is at reducing risk of sharing sensitive data across separate invited collaborators, it is not inherently a means to breaking down siloes.

Essential companions to the TRE are dynamically-updated and searchable metadata catalogs, *in situ* analysis tools with code versioning, as well as data provenance, and audit trails (Graham et al., 2022). Without these, a TRE is simply a safe place for a single project to be completed and then archived with limited usefulness for new projects or data consumers on other project teams. A DRE Workspace is a Trusted Research Environment–providing a safe-haven for clinical researchers, bioinformaticians and pharmacologists to analyze and develop models on sensitive data with the confidence that the data and models developed are secure and protected. A DRE builds on the concept of a TRE in that it provides remote access to data alongside tools for analysis in a securely controlled workspace, but it also adds essential components that allow the data and tools to be FAIR (Findable, Accessible, Interoperable, and Reusable), version-controlled and dynamically growing in size or quality as a result of each collaboration, and to break down the silos often created by aggregating and analyzing data as a single-use asset.

Vital to collaborative development is the need for individuals to modify, change and fix on their own versions of code without disrupting other team members. When code is committed and a submission is made, either to a research paper, as a thesis or for approval from a regulatory body–reproducibility of outcomes is essential. It must be possible to re-run the exact code, on the exact data on the exact compute infrastructure and verify that the results and outcomes are unchanged. Such transparent reproducibility is only possible with version control software.

**FIGURE 2:**
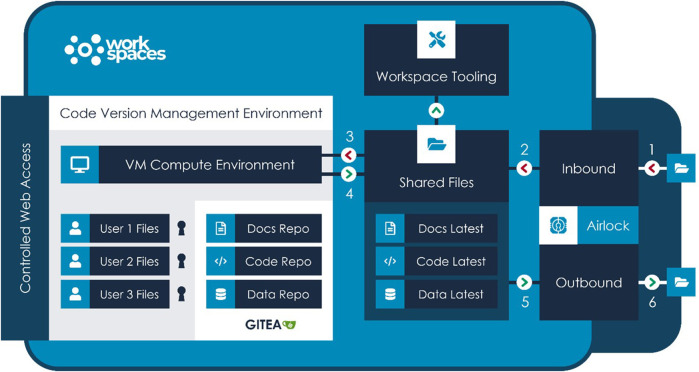
Diagram illustrating a typical flow for development and review purposes based on the Aridhia DRE Workspace Environment.

Outside of a workspace, Git -Scm (2023), the open-source versioning control software, along with GitHub (2023) for hosting has become the standard for collaborative development, not only for code but for API standards and, significantly for healthcare data science, ontologies. Figure 2 illustrates a typical flow for development and review purposes for the Aridhia DRE as an example of a DRE workspace environment. Important elements of the workflow are the security access to the DRE in general, the FAIR data services which manage the ingestion, curation and integration aspects of the process and provide audit trails to any further data transformation and the workspace tooling of both open-souce and proprietary software or connectivity to other platforms that could provide AI/ML solutions *via* access from the DRE.

Inside a workspace, where data is often highly sensitive, security is of paramount importance and access to online repositories is typically prohibited, users still require the Git-like abilities to create repositories, clone them, import external repositories (with appropriate review and security controls) and push reviewed and approved code to repositories or other workspaces as part of the outbound airlock process. For drug discovery purposes there are a number of open source databases that would provide an anchor for AI-based predictions but most commonly these data will be joined by proprietary data from drug sponsors seeking to protect their intellectual property. Best practice recommendations for code development and publishing with a Trusted Research Environment (Chalstrey, 2021) have been proposed by the Turing Institute.

The paper by the Turing Institute promotes best practice recommendations (Chalstrey, 2021). Key to the conclusions within the paper is the requirement to version code, data and compute within the scope of a DRE. Outcomes must be digitally reproducible and the paper recommends the use of Conda for package management and Git for code, model and (where appropriate) data versioning. The Aridhia DRE workspace provides support for the use of Anaconda (https://knowledgebase.aridhia.io/article/installing-anaconda-and-running-jupyter-notebooks-on-the-virtual-machine/) and has now released the integration of Gitea (https://gitea.io/en-us/). Users of a workspace now have access to their own completely secure version control system. This is completely locked within the scope of a Workspace, ensuring that data and code cannot leak to other workspaces or leave the workspace environment without going through the accepted airlock release checks and balances. Based on discussion with data science, modelling and governance communities, this approach seems to hit the right balance between freedom to operate for workspace owners and the rigorous security and information governance requirements of data controllers.

As it pertains to drug discovery in particular, historically most large PhRMA companies have relied on their own chemical libraries as sources for both target validation and candidate selection. This is limiting in the sense that this data defines therapeutic areas of current interest where historical libraries may be thin. To augment this deficit, occasionally companies may either develop partnerships with some organizations having more extensive libraries or acquire them entirely [BioProcess Online Press release. “ChemRx To Develop Compounds For Signal Pharma Under New Agreement,”1998, https://www.bioprocessonline.com/doc/chemrx-to-develop-compounds-for-signal-pharma-0001 and Astellas press release, “Astellas Announces Acquisition of Nanna,” 2020, https://www.astellas.com/en/news/15756] both of which are timely, costly and may have intellectual property (IP) considerations. This behaviour continues with a single goal in mind, to significantly extend capabilities to support drug discovery. More is definitely better in the case of chemical libraries. Even though this is often outside the realm of data sharing, the details of data acquisition, curation and integration are essential and often not exposed to any great extent and are likely to be time consuming and costly. It’s likewise a setting where an internally facing DRE may have an advantage over more traditional ETL (extract, transform, load) procedures. In the case where collaborations are in place (e.g., academic and industry based with IP sharing under contractual agreement), data sharing must be more directly dealt with and described in detail in both data collaboration and data use agreements (DUAs and DCAs) (Barrett, 2020). The data sharing application of both TREs and DREs are obvious with many examples including Yoda (Ross et al., 2018), RDCA-DAP (Larkindale et al., 2022), ADDI (ADDI, 2021) and ICODA (Zariffa et al., 2021) to name a few. Some focus on providing honest broker services for data designated for public sharing (Yoda) and others emphasize a collaborative research environment from which data sharing and collaboration *via* analytic tool development (RDCA-DAP) can actually occur.

## AI/ML–Drug discovery use cases

There are many open-source projects which have been creating collaborative tooling for drug discovery (e.g., VirtualFlow, https://github.com/VirtualFlow/VFVS). The opportunity for different drug discovery libraries to be used within a DRE can further aid in creating a trustworthy, transparent, and collaborative research environment. Scarcity of biological data for the purpose of building accurate and robust Machine learning models in the area of drug discovery is a well-known problem. Open source tools and projects are providing a solution through the additional tooling and collaborative approach to addressing this problem. Below we identify several, mostly academic efforts to guide and support drug discovery in targeted therapeutic areas where AI-based approaches have been integrated into drug discovery milestones. The use cases are reviewed highlighting their focus and track record in advancing drug discovery science. We also provide and assessment for how these use cases could be further optimized in a DRE environment.

### Drug discovery for TB combination therapy—TUFTS university

Until COVID-19 appeared in 2020, tuberculosis (TB) was acknowledged as the deadliest infectious disease and still tops the list of deadly infectious diseases (European Centre for Disease Prevention and Control Press Release, 2022). Its current therapeutic treatment is based on a cocktail of four drugs taken for 4 months duration with significant side effects that make it difficult for many patients to complete the regimen. TB is also becoming increasingly resistant to standard treatments. The TUFTS research environment and platform creates accurate predictions of how effective treatments will perform when moving from testing in a lab to testing in mouse models which is typically the purview of drug discovery. Promising new antibiotics aimed at TB have been developed recently. But researchers and drug developers have been challenged to find ways to cheaply and effectively determine which drugs will work best in combination (Terreni et al., 2021). It’s a mathematically complex, expensive, and time-consuming process using traditional procedures.

During drug development, scientists first test potential pharmaceuticals in the lab relying on *in vitro* experiments with TB bacteria. Treatments that kill TB proceed to testing in lab rodents, and those regimens that are determined effective in rodents proceed eventually to clinical trials in humans. Combination therapy is necessary because the TB bacterium *Mycobacterium tuberculosis* is highly adaptive to its site of infection, giving rise to differences in drug response among the bacteria in a single person. Also, individuals may be infected with a drug-resistant strain, or their TB infection may evolve over time to become resistant to one or more of the traditional cocktail’s antibiotics. Using mathematical models and artificial intelligence, the Tufts team discovered a set of rules that drug pairs need to satisfy to be potentially good treatments (Aldridge et al., 2021).

The AI predictive algorithm DiaMOND (Larkins-Ford et al., 2022) is based on diagonal measurement of n-way drug interactions, a method to systemically study pairwise and high-order drug combination interactions to identify shorter, more efficient treatment regimens for TB and diseases that require combination drug therapy. With design rules established, researchers believe this system can increase the speed at which discovery scientists can determine which drug combinations will most effectively treat tuberculosis. This modeling system and the use of drug pairs (rather than combinations of three or four drugs) cuts down significantly on the amount of testing that needs to be done before moving a drug pair into further study. As this system becomes an option for drug developers, mechanisms to deal with varied data contributors and data governance/provenance issues may necessitate the evolution of this platform to become a multi-stakeholder DRE. At the moment, interested stakeholders can contact the PI for interest in supplying drug for experimental and *in silico* analysis with potential combination outcomes generated in an iterative manner. External stakeholders cannot access the environment directly and are reliant on the research team to publish findings to generate their own summary data for subsequent analyses.

### DeepChem and TorchDrug and other chemical library examples

Another project aiming to create collaborative open-source tools for drug discovery as well as material science, quantum chemistry and biology is the DeepChem project (DeepChem, 2022). The project aims to address some of the key issues when building machine learning models on molecules, namely, limited amount of data, wide range of outputs to predict, large heterogeneity in input molecular structure and appropriate learning algorithms to benchmark model performance (Wu et al., 2018). The paper “Low Data Drug Discovery with one-shot learning” points out the central problem of small-molecule based drug discovery is to optimize the candidate molecule by finding analogue molecules with increased pharmaceutical activity and reduced patient risks. The paper outlines the capacity for deep neural networks is underpinned by their ability to learn from large amounts of biological data. The lack of large datasets for models to learn and form accurate predictions for novel compounds remains a challenge in drug discovery. Using the DeepChem open-source framework for deep-learning they demonstrate how one-shot learning can lower the amount of data required to make meaningful predictions in drug discovery (Altae-Tran et al., 2017). The DeepChem library was extended into an end-to-end modelling pipeline for drug discovery through the ATOM Modeling PipeLine [AMPL] (Minnich et al., 2020). AMPL showed DeepChem can be used in a large scale pipeline for Pharma drug discovery efforts.

Build on top of the PyTorch library, TorchDrug benchmarks a variety of important tasks in drug discovery, including molecular property prediction, pretrained molecular representations, *de novo* molecular design and optimization, retrosynthsis prediction, and biomedical knowledge graph reasoning (Zhu et al., 2022). Therapeutics Data Commons (TDC) a platform comprised of three components, namely, 66 AI-ready datasets and 22 learning tasks for drug discovery and development, an ecosystem of tools and community resources and leaderboards for therapeutics machine learning (Huang et al., 2022). In the context of relational deep learning, ChemicalX (Rozemberczki et al., 2022) is a deep learning library for drug-drug interactions, polypharmacy side effect, and synergy predictions. Its deep neural network architecture can be used to solve the drug pair scoring task. By predicting the outcome of administering two drugs together in a biological or chemical context (ChemicalX, 2022). In the area of genomic deep learning, a tool that is envisioned to facilitate drug discovery is Graphein (Jamasb et al., 2020). It is a tool for transforming raw data from widely-used bioinformatics databases into machine learning-ready datasets. It is a Python library for constructing graph and surface-mesh representations of protein structures and biological interaction networks for computational analysis. The use cases in this example illustrate how chemical libraries can be interfaced with AI-based tooling to answer early phase drug development questions. The missing element in these efforts in the appropriateness of the various “AI-ready datasets” described above to certain, specific contexts of use across a variety of target therapeutic areas and drug targets. Likewise, the incremental benefit of augmented such data with data from a drug sponsor is not known. Both of these aspects could be accommodated within the confines of a sponsor-governed and owned DRE in which the such libraries could co-exist and in which software and tooling could be directly deployed within a DRE workspace or run within a virtual machine that is deployed in a DRE workspace.

### DRE requirements for AI/ML data exploitation and collaboration

#### Data requirements

Despite some misconceptions about the ability to simply drop an army of messy data into a generic file repository, run some algorithms, and receive magical insights, data requirements for efficient, effective AI and ML models are significant (Paullada et al., 2021). When biotech companies outsource AI projects in the drug discovery phase, they often ship their proprietary data off to external vendor platforms to be transformed and standardized as needed for aggregation with subsets from open-source data repositories. In addition to the risk of data leakage, companies lose visibility into the data curation work and may have a loose connection to its provenance. This situation creates potential for unintended costs in time, insights, and money in several ways. Different vendor teams may be repeating the same data cleaning each time and charging proportionately, or they may choose to leave data as it is received but charge for the excessive compute resources needed to analyze messy data. In some cases, messy data give insights but take longer to process, and in other cases the messy data produce noisy and unhelpful mathematical results.

Moreover, companies’ transformed data are not useful for future projects with new formats or model input specifications, which also fails to extract the full potential of the data for later phases of the drug development process. An alternative that solves these concerns is keeping proprietary data in a private company DRE where data transformations and other cleaning processes may be standardized across projects with audit trails to provide provenance and a robust metadata catalog to increase interoperability and reusability. Owning internal data curation standards allows federated access to open-source repositories by maintaining the interoperability of internal data assets. The DRE in this case reduces the multiplying cost of sending data to outsourced vendors to be repeatedly processed, but it also facilitates dynamic improvement of data assets for all projects in the organization. Because the DRE provides internal AI/ML experts and external contractors secure access to data, tools and scalable compute resources in one audited environment, the downstream advantage is that companies may use their proprietary data assets in later phases of drug development where regulatory tolerance for data and algorithms without provenance is minimal.

#### Compute requirements

The DRE, as the research environment, requires access to appropriately sized and configured pools of compute. Depending on the tools used and environment required to run them, these can range from dedicated machines, such as a Virtual Machine or “Instance” in a cloud environment to High Performance Compute (HPC) clusters dedicated to running batch systems, where discrete jobs can be run across a cluster in parallel, allowing for distributed compute and processing. These batch processing environments will orchestrate the execution of jobs in accordance with developer configuration, to produce optimal results. These systems can significantly reduce the time to execute ML processes, however, can often be cost prohibitive. An alternative to a dedicated HPC cluster is to take advantage of cloud services offered by all the major cloud vendors such as AWS, Azure and Google. Each has ML focused batch compute environments that can be run on demand using only the compute they need. Developers and researchers can choose the balance between runtime and costs and the system ensures that only the compute required is run at any point in time.

As learning algorithms need to run against as many different training sources as possible, potentially across various geographies, the need to federate this compute across multiple environments becomes more pertinent. The Global Alliance For Genomic Health has developed a standard application programming interface (API) known as the Task Execution Service (TES). The TES provides a standard API for nodes of a federated network to implement, allowing for an orchestrated machine learning system to execute jobs relevant to their tasks on remote compute nodes. This can ensure that data remains within the geography and within the DRE implementing the API. The results of the execution are provided to the orchestration engine to inform the learning process.

## Discussion

AI/ML techniques have been successfully applied to drug discovery and early stage drug development but most of the successful use cases have been generated through partnerships and outsourced solutions (Liu et al., 2021; Terreni et al., 2021; Schauperl and Denny, 2022). The benefit of TRE or even DRE solutions has been less appreciated as they appear to create a sense of vulnerability from an intellectual property standpoint as data sharing is the presumed emphasis of such solutions. Likewise, FAIR and workspace services are appreciated for late stage drug development but don’t always seem essential for drug discovery and late stage development based on a presumed high overhead (effort and cost). The reality however is that AI-based drug discovery applications can be more easily managed and controlled in a robust, DIY (do-it-yourself) approach which cannot only provide an integrated solution owned and managed by the sponsor but also offer a complementary solution to established MIDD approaches that facilitate decision making.

Data sharing initiative are many including many consortia (e.g., https://www.melloddy.eu/, https://www.pistoiaalliance.org/, etc.) but in fact the majority of these are focused on late-stage drug development and the sharing of clinical trial, patient-level data and not early-stage development or drug discovery data. Other initiatives such as the Open Reaction Database (https://docs.open-reaction-database.org/en/latest/index.html) are more complementary as they seek to build an open access chemical reaction database to support machine learning and related efforts in reaction prediction, chemical synthesis planning, and experiment design. A consortium-based approach to accumulating and sharing drug discovery data is unlikely given concerns for IP as defined previously.

The MIDD approach is well entrenched in the current drug development paradigm both from innovators seeking to develop new drugs and new drug targets as well as regulators who must judge the suitability of evidentiary proof that proposed new treatments are both safe and efficacious. The current approach is highly dependent on quantitative scientists with expertise in various forms of modeling and simulation methodologies, a suite of software solutions that permit the development, codification, and validation of discrete models that de-risk decision making and a modern compute environment to house the software and model libraries so that such resources can be maintained in a secure, Part 11 compliant manner and share among its practitioners. Most importantly, the current MIDD approach relies on buy-in from senior leaders within and organization and favorable interactions with regulatory authorities. Likewise, as the approach evolves and certainly if considerations for an AI-enabled approach is put forward, these interactions will require revisiting with additional education and performance confirmation.

The field is constantly evolving requiring additional skillsets and expertise as well as diverse software solutions, customized and secure compute platforms and new methodologies and approaches. Sponsors likewise recognize that investment in MIDD is similarly evolving and growing. As the industry constantly seeks more efficient and cost-effective solutions, MIDD is not immune to this scrutiny. Much of the recent effort to improve the efficiency of MIDD is based on improved access to integrated data sources, creating libraries of model elements that can be shared and combined as needed and improving the compute platform to ensure that relevant tools and software solutions can coexist on the same platform. Little has been done to alter the data or model types that inform the commonly held critical decisions as previously outlined.

Artificial intelligence has received much interest of late as a complementary tool to answer specific drug development questions and pharmaceutical sponsors have been both dedicating internal resources and investing in external partnerships to enhance their knowledge and expertise in this discipline (Liu et al., 2020). An outgrowth of this interest has been the submission of some of these efforts to regulatory authorities. As recent work suggests (Liu et al., 2023), the number of these submissions has increased dramatically recently allowing authorities to gain an understanding of the diversity of applications that might support drug development and judge the usefulness of the approach for regulatory decision making. Some positive outcomes from this early experience includes the generation of early thoughts on guiding principles and the development of consortiums and shared resources. While these are early days in the process of getting comfortable with the approach and gaining confidence in the application, it is also an opportunity to gage future requirements for submission of such AI/ML implementations and consider how transformative the approach could be in a more coordinated manner. Early regulatory guidance (Liu et al., 2020; Liu et al., 2023) suggest that regulatory authorities are also anticipating broader utilization.

A future, but hopefully near-term effort should include the consideration of AI/ML application as either a complimentary or entirely self-sufficient approach to support an MIDD paradigm, particularly in support of early stage drug development. Early adopters of the approach tend to compartmentalize the effort into certain drug development sectors (Liu et al., 2020; Maharao et al., 2020; Paul et al., 2021) but few have considered this as an end-to-end solution. Moreover, the approach is still being compared to traditional MIDD efforts, specifically around the comparison of AI/ML prediction against specific model types (PBPK, PK/PD, CTS, etc.) and not around the data types and dimensions that would inform the various approaches or whether a revamped approach considering the optimal information value (driven by data and models) needed to guide regulatory milestones. While drug discovery and early stage drug development may be considered an acceptable frontier for this effort at face value given the presumed lower bar for regulatory buy-in and acceptance, there will be continuity gains for ensuring data traceability and audit trails as the early-stage AI deliverables become linked to later-stage efforts and milestones. Clearly, the path forward involves collaboration and an open mind with respect to optimized and informative data generation coupled with tools that can be utilized with high fidelity based on mutually agreed and objective performance criteria.

## Data Availability

The original contributions presented in the study are included in the article/supplementary materials, further inquiries can be directed to the corresponding author.
